# Association between green space exposure and elderly health: a systematic review and meta-analysis

**DOI:** 10.1186/s12889-025-26137-y

**Published:** 2026-01-08

**Authors:** Mengyao Wang, Yajie Che, Xinyun Tan, Nan Zhang, Shan Yu, Ping Yan

**Affiliations:** 1 School of Nursing, Xinjiang Medical University, Urumqi, Xinjiang Uygur Autonomous Region 830000 China; 2 Health Care Research Center for the Xinjiang Regional Population, Urumqi, Xinjiang Uygur Autonomous Region 830000 China; 3 Xiangya School of Nursing, Central South University, Changsha, Hunan 410083 China; 4 Xinjiang Uygur Autonomous Region, The Fifth Affiliated Hospital of Xinjiang Medical University, Urumqi, 830011 China

**Keywords:** Green space exposure, Elderly health, Systematic review

## Abstract

**Background:**

Exposure to green spaces is associated with many health benefits across various stages of life. However, no comprehensive synthesis currently exists to consolidate this evidence into a systematic body of knowledge that captures the overall impact of exposure to green space on the health of older adults. This systematic review seeks to address this gap by generating a thorough, integrated evidence base and offering valuable insights for future research directions and practical applications.

**Methods:**

We adopted an extensive search strategy, drawing from multiple electronic databases as well as the National Institute for Health and Clinical Excellence (United Kingdom) and the Guidelines International Network. The electronic databases searched included the Cochrane Library, Medline, Embase, CINAHL, PubMed, Web of Science, Scopus, Global Health, and CNKI, using the PEOS search framework. The search encompassed publications from the inception of each database to November 2024. A total of 5,749 records were initially identified, and, following a dual-reviewer screening and selection process, 27 studies were ultimately included in the review.

**Results:**

Exposure to green spaces was associated with effects on circulatory system disorders, mental health conditions, nervous system diseases, cognitive decline, metabolic disorders, and overall life satisfaction in older adults. Evidence regarding cardiovascular outcomes was mixed, with some studies indicating a U-shaped relationship for hypertension. Exposure to green space demonstrated a protective role against metabolic diseases and was positively associated with enhanced life satisfaction among older adults. Meta-analyses revealed that exposure to green space was associated with a lower risk of Alzheimer’s disease ( OR= 0.856, 95% CI: 0.769–0.943) and depression (OR= 0.724, 95% CI: 0.549–0.900).

**Conclusions:**

This systematic review synthesizes the multifaceted health effects of green environments on ageing populations, critically evaluating empirical evidence on exposure to green space and health outcomes in older adults. The findings present a robust, evidence-based framework that underscores consistent associations between the accessibility and quality of green spaces and key indicators of geriatric health, thereby laying the groundwork for designing targeted, nature-based behavioral interventions.

**Trial registration:**

PROSPERO (CRD42024619700).

**Supplementary Information:**

The online version contains supplementary material available at 10.1186/s12889-025-26137-y.

## Introduction

Green spaces encompass land areas covered with natural vegetation, parks, residential gardens or courtyards, and green infrastructure [1]. They represent an essential component of urban environments, fostering comfortable and relaxing surroundings. These spaces create favorable social conditions for residents, encouraging physical activity and opportunities to connect with nature, thereby generating substantial socioeconomic benefits [2]. Exposure to green space refers to the degree of interaction between urban populations and vegetated areas, typically measured across three main dimensions: (1) availability (total area), (2) accessibility (proximity metrics), and (3) quality (aesthetic and functional attributes). Collectively, these spatially explicit indicators evaluate exposure through geometric accessibility (Euclidean or network distances), spatial visibility (viewshed analysis), and service capacity (facility completeness) [3]. The vegetation index—most commonly the Normalized Difference Vegetation Index (NDVI)—is calculated by comparing remote sensing reflectance values in the near-infrared and red light bands and is used to evaluate vegetation density. Exposure to green spaces can provide health benefits across all stages of life. A higher NDVI level has been associated with a lower risk of congenital heart disease in fetuses and is positively correlated to increased neonatal birth weight [4–7]. Exposure to green space offers multiple advantages for children’s health, including a significant reduction in the risk of respiratory and allergic diseases, as well as a decreased likelihood of developing myopia [8–12]. Epidemiological evidence further suggests that prenatal exposure to green spaces is associated with a reduced risk of pregnancy-associated hypertensive disorders and gestational diabetes mellitus, while also conferring positive maternal–fetal outcomes [13–15]. Cognitive health benefits have also been observed in diverse age groups [16]. Specifically, exposure to green space has been associated with a lower risk of attention deficit hyperactivity disorder in children, enhanced attention capacity in adults, and a reduced risk of dementia in older adults [16]. In older populations, evidence indicates a correlation between exposure to green space and decreased incidence rates of chronic conditions such as cardiovascular diseases (CVDs), neurodegenerative disorders, metabolic syndrome (MetS), and psychological distress [17–19]. Taken together, these findings highlight the life-course health promotion potential of green infrastructure, positioning it as a cost-effective preventive measure for managing multimorbidity and supporting healthy aging in urban settings.

With the global population increasingly concentrated in cities, projections estimate that by 2050, urban residents will constitute 68% of the total population [20]. Against the backdrop of concurrent global population aging and rapid urbanization, the health effects of exposure to green space in older adults have become a prominent area of research [21]. However, to our knowledge, no systematic review has yet comprehensively examined the overall health effects of exposure to green space in this demographic. This review aims to address this gap by answering the following research questions: Is exposure to green space associated with health outcomes in older adults? Which specific aspects of older adults’ health are influenced by such exposure? The present study seeks to provide a thorough and integrated evidence base to inform future research and guide practical applications.

## Methods

This systematic review was conducted in accordance with a prespecified protocol and the PEOS framework (Population, Exposure, Outcome, and Study Design) [22]. The protocol has been registered in the International Prospective Register of Systematic Reviews (PROSPERO; registration number: CRD42024619700) and the report adheres to the Preferred Reporting Items for Systematic Reviews and Meta-Analysis (PRISMA 2020) guidelines.

### Eligibility and selection criteria

Studies that met the following criteria were included in this systematic review: (1) Population: participants were people aged ≥ 60 years in the community. (2) Exposure: Exposure to green space was evaluated from three aspects: the degree of exposure to green spaces (used NDVI), the types of greenery (such as parks, vegetated areas, forests, or open green spaces), and the green space accessibility (older adults self-reporting). (3) Outcome: Common chronic physical and mental diseases among older people [23], including respiratory tract diseases (such as bronchitis, asthma, rhinitis allergic, chronic obstructive pulmonary disease), cardiovascular system diseases (such as coronary heart disease and hypertension), metabolic disorders (such as diabetes, osteoporosis, and dyslipidemias), nervous system diseases (such as stroke, sarcopenia, and sleep wake disorders), mental disorders (such as anxiety, depression and schizophrenia, and cognitive impairments), isolation, life satisfaction, and subjective well-being. (4)All observational studies (primarily cohort and cross-sectional studies), experimental studies, and both published or unpublished grey literature were included. We excluded the review and animal experiment. Language was not restricted in this research.

### Search strategy

In order to comprehensively identify the relevant literature, we implemented a comprehensive search strategy using electronic databases, National Institute for Health and Clinical Excellence of the United Kingdom, and Guidelines International Network. The electronic databases included Cochrane Library, Medline, Embase, CINAHL, PubMed, Web of Science, Scopus, Global Health, and CNKI. The search time range was from the time of establishment of each database until November 2024. The detailed search strategy is presented in Table 1.

**Table 1:** Search strategy

Database	Search strategy	Search
PubMed	#1 Greenspace (((Parks, Recreational[MeSH Terms]) OR (((((((((((((((((((((((((((((((((Parks, Recreational[Title/Abstract]) OR (Park, Recreational[Title/Abstract])) OR (Recreational Park[Title/Abstract])) OR (Recreational Parks[Title/Abstract])) OR (Urban Parks[Title/Abstract])) OR (Parks, Urban[Title/Abstract])) OR (Park, Urban[Title/Abstract])) OR (Urban Park[Title/Abstract])) OR (National Parks[Title/Abstract])) OR (National Park[Title/Abstract])) OR (Park, National[Title/Abstract])) OR (Parks, National[Title/Abstract])) OR (Community Parks[Title/Abstract])) OR (Community Park[Title/Abstract])) OR (Park, Community[Title/Abstract])) OR (Parks, Community[Title/Abstract])) OR (Green Space[Title/Abstract])) OR (Green Spaces[Title/Abstract])) OR (Space, Green[Title/Abstract])) OR (Greenness[Title/Abstract])) OR (green infrastructure[Title/Abstract])) OR (Wilderness[Title/Abstract])) OR (wild land[Title/Abstract])) OR (natural land[Title/Abstract])) OR (community land[Title/Abstract])) OR (natural space[Title/Abstract])) OR (Park[Title/Abstract])) OR (park access[Title/Abstract])) OR (wild area[Title/Abstract])) OR (natural area*[Title/Abstract])) OR (green area*[Title/Abstract])) OR (NDVI[Title/Abstract])) OR (Normalized Difference Vegetation Index[Title/Abstract]))) #2:Aged (("Aged"[Mesh]) OR (((((((((((Aged[Title/Abstract])) OR (elderly[Title/Abstract])) OR (older adult[Title/Abstract])) OR (old people[Title/Abstract])) OR (old person[Title/Abstract])) OR (Elderly people[Title/Abstract])) OR (elderly person[Title/Abstract])) OR (elderly population[Title/Abstract])) OR (seniors[Title/Abstract])))) #3:Respiratory Tract Diseases ((((((("Respiratory Tract Diseases"[Mesh]) OR (((((((((Respiratory Tract Diseases[Title/Abstract]) OR (Bronchial Diseases[Title/Abstract])) OR (Asthma[Title/Abstract])) OR (Bronchitis[Title/Abstract])) OR (Bronchitis, Chronic[Title/Abstract])) OR (Rhinitis, Allergic[Title/Abstract])) OR (chronic obstructive pulmonary disease[Title/Abstract])) OR (COPD[Title/Abstract])) OR (copd[Title/Abstract]))) #4:Cardiovascular Diseases (("Cardiovascular Diseases"[Mesh]) OR (((((cardiovascular system diseases[Title/Abstract]) OR (heart diseases[Title/Abstract])) OR (Coronary Vessel Anomalies[Title/Abstract])) OR (coronary heart disease[Title/Abstract])) OR (hypertension[Title/Abstract])))) #5:Metabolic Diseases (("Metabolic Diseases"[Mesh]) OR ((((((Metabolic Diseases[Title/Abstract]) OR (Osteoporoses[Title/Abstract])) OR (Senile Osteoporoses[Title/Abstract])) OR (Diabetes Mellitus[Title/Abstract])) OR (Dyslipidemias[Title/Abstract])) OR (Hyperlipidemias[Title/Abstract])))) #6:Nervous System Diseases (("Nervous System Diseases"[Mesh]) OR (((((Nervous System Diseases[Title/Abstract]) OR (stroke[Title/Abstract])) OR (Sleep Wake Disorders[Title/Abstract])) OR (Sleep Deprivation[Title/Abstract])) OR (sarcopenia[Title/Abstract])))) #7:Mental Disorders (("Mental Disorders"[Mesh]) OR (((((((((((((Mental Disorders[Title/Abstract]) OR (Anxiety Disorders[Title/Abstract])) OR (Anxiety Neuroses[Title/Abstract])) OR (Bipolar[Title/Abstract] AND Related Disorders[Title/Abstract])) OR (Depressive Disorder[Title/Abstract])) OR (Depressive Neuroses[Title/Abstract])) OR (Depressive Syndrome[Title/Abstract])) OR (Psychotic Disorders[Title/Abstract])) OR (Cognitive Dysfunction[Title/Abstract])) OR (Mild Cognitive Impairment[Title/Abstract])) OR (Cognitive Decline[Title/Abstract])) OR (Psychoses[Title/Abstract])) OR (Schizoaffective Disorder[Title/Abstract])))) #8:life satisfaction ((((isolation[Title/Abstract]) OR (life satisfaction[Title/Abstract])) OR (Personal Satisfaction[Title/Abstract])) OR (Subjective Well-Being[Title/Abstract]))) #9:#3OR #4OR #5OR #6OR #7OR #8 #1 AND #2 AND #9	990
EMBASE	#1 Greenspace 'Parks, Recreational*':ab,ti OR 'Recreational Park*':ab,ti OR 'Park, Urban*':ab,ti OR 'Urban Park*':ab,ti OR 'National Park*':ab,ti OR 'Park, National*':ab,ti OR 'Community Park*':ab,ti OR 'Park, Community*':ab,ti OR 'Green Space*':ab,ti OR 'Space, Green*':ab,ti OR 'Greenness':ab,ti OR 'green infrastructure*':ab,ti OR 'Wilderness':ab,ti OR 'wild land*':ab,ti OR 'natural land*':ab,ti OR 'community land*':ab,ti OR 'natural space*':ab,ti OR 'Park':ab,ti OR 'park access*':ab,ti OR 'wild area*':ab,ti OR 'natural area*':ab,ti OR 'green area*':ab,ti OR 'NDVI':ab,ti OR 'Normalized Difference Vegetation Index*':ab,ti #2: Aged 'Aged':ab,ti OR 'Elderly':ab,ti OR 'older adult*':ab,ti OR 'old people*':ab,ti OR 'old person*':ab,ti OR 'Elderly people*':ab,ti OR 'elderly person*':ab,ti OR 'elderly population*':ab,ti OR 'Seniors':ab,ti #3:Respiratory Tract Diseases 'Respiratory Tract Diseases*':ab,ti OR 'Bronchial Diseases*':ab,ti OR 'Asthma':ab,ti OR 'Bronchitis':ab,ti OR 'Bronchitis, Chronic*':ab,ti OR 'Rhinitis, Allergic*':ab,ti OR 'chronic obstructive pulmonary disease*':ab,ti OR 'COPD':ab,ti OR 'Copd':ab,ti #4:Cardiovascular Diseases 'Cardiovascular Diseases*':ab,ti OR 'cardiovascular system diseases*':ab,ti OR 'heart diseases*':ab,ti OR 'Coronary Vessel Anomalies*':ab,ti OR 'coronary heart disease*':ab,ti OR 'Hypertension':ab,ti #5:Metabolic Diseases 'Metabolic Diseases*':ab,ti OR 'Osteoporoses':ab,ti OR 'Senile Osteoporoses':ab,ti OR 'Diabetes Mellitus*':ab,ti OR 'Dyslipidemias':ab,ti OR 'Hyperlipidemias':ab,ti #6:Nervous System Diseases 'Nervous System Diseases*':ab,ti OR 'Stroke':ab,ti OR 'Sleep Wake Disorders*':ab,ti OR 'Sleep Deprivation*':ab,ti OR 'Sarcopenia':ab,ti #7:Mental Disorders 'Mental Disorders*':ab,ti OR 'Anxiety Disorders*':ab,ti OR 'Anxiety Neuroses*':ab,ti OR 'Bipolar and Related Disorders*':ab,ti OR 'Depressive Disorder*':ab,ti OR 'Depressive Neuroses*':ab,ti OR 'Depressive Syndrome*':ab,ti OR 'Psychotic Disorders*':ab,ti OR 'Cognitive Dysfunction*':ab,ti OR 'Mild Cognitive Impairment*':ab,ti OR 'Cognitive Decline*':ab,ti OR 'Psychoses':ab,ti OR 'Schizoaffective Disorder*':ab,ti #8:life satisfaction 'isolation':ab,ti OR 'life satisfaction*':ab,ti OR 'Personal Satisfaction*':ab,ti OR 'Subjective Well-Being*':ab,ti #9:#3OR #4OR #5OR #6OR #7OR #8 #1 AND #2 AND #9	714
WoS	#1 Greenspace ((((((((((((((((((TS = (Park)) OR TS = (Recreational Park)) OR TS = (Urban Park)) OR TS = (National Park)) OR TS = (Community Park)) OR TS = (Green Space)) OR TS = (Greenness)) OR TS = (green infrastructure)) OR TS = (Wilderness)) OR TS = (wild land)) OR TS = (natural land)) OR TS = (community land)) OR TS = (natural space)) OR TS = (park access)) OR TS = (wild area)) OR TS = (natural area*)) OR TS = (green area*)) OR TS = (NDVI)) OR TS = (Normalized Difference Vegetation Index) #2: Diabetes Mellitus, type 2 ((((((((TS = (Aged)) OR TS = (elderly)) OR TS = (older adult)) OR TS = (old people)) OR TS = (old person)) OR TS = (elderly people)) OR TS = (elderly person)) OR TS = (elderly population)) OR TS = (seniors) #3:Respiratory Tract Diseases ((((((((TS = (Respiratory Tract Diseases)) OR TS = (Bronchial Diseases)) OR TS = (Asthma)) OR TS = (Bronchitis)) OR TS = (Bronchitis Chronic)) OR TS = (Rhinitis Allergic)) OR TS = (chronic obstructive pulmonary disease)) OR TS = (COPD)) OR TS = (copd) #4:Cardiovascular Diseases ((((TS = (cardiovascular system diseases)) OR TS = (heart diseases)) OR TS = (Coronary Vessel Anomalies)) OR TS = (coronary heart disease)) OR TS = (hypertension) #5:Metabolic Diseases (((((TS = (Metabolic Diseases)) OR TS = (Osteoporoses)) OR TS = (Senile Osteoporoses)) OR TS = (Diabetes Mellitus)) OR TS = (Dyslipidemias)) OR TS = (Hyperlipidemias) #6:Nervous System Diseases ((((TS = (Nervous System Diseases)) OR TS = (stroke)) OR TS = (Sleep Wake Disorders)) OR TS = (Sleep Deprivation)) OR TS = (sarcopenia) #7:Mental Disorders ((((((((((((TS = (Mental Disorders)) OR TS = (Anxiety Disorders)) OR TS = (Anxiety Neuroses)) OR TS = (Bipolar and Related Disorders)) OR TS = (Depressive Disorder)) OR TS = (Depressive Neuroses)) OR TS = (Depressive Syndrome)) OR TS = (Psychotic Disorders)) OR TS = (Cognitive Dysfunction)) OR TS = (Mild Cognitive Impairment)) OR TS = (Cognitive Decline)) OR TS = (Psychoses)) OR TS = (Schizoaffective Disorder) #8:life satisfaction (((TS = (isolation)) OR TS = (life satisfaction)) OR TS = (Personal Satisfaction)) OR TS = (Subjective Well-Being) #9:#3OR #4OR #5OR #6OR #7OR #8 #1 AND #2 AND #9	3359
Cochrane Library	#1 MeSH descriptor: [Parks, Recreational] explode all trees #2 (Parks, Recreational):ti,ab,kw OR (Park, Recreational):ti,ab,kw OR (Recreational Park):ti,ab,kw OR (Recreational Parks):ti,ab,kw OR (Urban Parks):ti,ab,kw OR (Parks, Urban):ti,ab,kw OR (Park, Urban):ti,ab,kw OR (Urban Park):ti,ab,kw OR (National Parks):ti,ab,kw OR (National Park):ti,ab,kw OR (Park, National):ti,ab,kw OR (Parks, National):ti,ab,kw OR (Community Parks):ti,ab,kw OR (Community Park):ti,ab,kw OR (Park, Community):ti,ab,kw OR (Parks, Community):ti,ab,kw OR (Green Space):ti,ab,kw OR (Green Spaces):ti,ab,kw OR (Space, Green):ti,ab,kw OR (Greenness):ti,ab,kw OR (green infrastructure):ti,ab,kw OR (Wilderness):ti,ab,kw OR (wild land):ti,ab,kw OR (natural land):ti,ab,kw OR (community land):ti,ab,kw OR (natural space):ti,ab,kw OR (Park):ti,ab,kw OR (park access):ti,ab,kw OR (wild area):ti,ab,kw OR (natural area*):ti,ab,kw OR (green area*):ti,ab,kw OR (NDVI):ti,ab,kw OR (Normalized Difference Vegetation Index):ti,ab,kw #3 #1 OR #2 #4 MeSH descriptor: [Aged] explode all trees #5 (Aged):ti,ab,kw OR (elderly):ti,ab,kw OR (older adult):ti,ab,kw OR (old people):ti,ab,kw OR (old person):ti,ab,kw OR (Elderly people):ti,ab,kw OR (elderly person):ti,ab,kw OR (elderly population):ti,ab,kw OR (seniors):ti,ab,kw #6 #4 OR #5 #7 MeSH descriptor: [Respiratory Tract Diseases] explode all trees #8 (Respiratory Tract Diseases):ti,ab,kw OR (Bronchial Diseases):ti,ab,kw OR (Asthma):ti,ab,kw OR (Bronchitis):ti,ab,kw OR (Bronchitis, Chronic):ti,ab,kw OR (Rhinitis, Allergic):ti,ab,kw OR (chronic obstructive pulmonary disease):ti,ab,kw OR (COPD):ti,ab,kw OR (copd):ti,ab,kw #9 #7 OR #8 #10 MeSH descriptor: [Cardiovascular Diseases] explode all trees #11 (cardiovascular system diseases):ti,ab,kw OR (heart diseases):ti,ab,kw OR (Coronary Vessel Anomalies):ti,ab,kw OR (coronary heart disease):ti,ab,kw OR (hypertension):ti,ab,kw #12 #10 OR #11 #13 MeSH descriptor: [Metabolic Diseases] explode all trees #14 (Metabolic Diseases):ti,ab,kw OR (Osteoporoses):ti,ab,kw OR (Senile Osteoporoses):ti,ab,kw OR (Diabetes Mellitus):ti,ab,kw OR (Dyslipidemias):ti,ab,kw OR (Hyperlipidemias):ti,ab,kw #15 #13 OR #14 #16 MeSH descriptor: [Nervous System Diseases] explode all trees #17 (Nervous System Diseases):ti,ab,kw OR (stroke):ti,ab,kw OR (Sleep Wake Disorders):ti,ab,kw OR (Sleep Deprivation):ti,ab,kw OR (sarcopenia):ti,ab,kw #18 #16 OR #17 #19 MeSH descriptor: [Mental Disorders] explode all trees #20 (Mental Disorders):ti,ab,kw OR (Anxiety Disorders):ti,ab,kw OR (Anxiety Neuroses):ti,ab,kw OR (Bipolar and Related Disorders):ti,ab,kw OR (Depressive Disorder):ti,ab,kw OR (Depressive Neuroses):ti,ab,kw OR (Depressive Syndrome):ti,ab,kw OR (Psychotic Disorders):ti,ab,kw OR (Cognitive Dysfunction):ti,ab,kw OR (Mild Cognitive Impairment):ti,ab,kw OR (Cognitive Decline):ti,ab,kw OR (Psychoses):ti,ab,kw OR (Schizoaffective Disorder):ti,ab,kw #21 #19 OR #20 #22 (isolation):ti,ab,kw OR (life satisfaction):ti,ab,kw OR (Personal Satisfaction):ti,ab,kw OR (Subjective Well-Being):ti,ab,kw #23 #9 OR #12 OR #15 OR #18 OR #21 OR #22 #3 AND #6 AND #23	640
Global health	"Parks, Recreational" AND "Aged" AND ("Respiratory Tract Diseases" OR "Cardiovascular Diseases" OR "Metabolic Diseases" OR "Nervous System Diseases" OR "Mental Disorders" OR "Personal Satisfaction")	33

### Risk of bias assessment

Two researchers (MY W and YJ C) independently evaluated the quality of all included literature. The risk of bias in randomized controlled trials (RCTs) was assessed using the Cochrane Handbook V.5.1.0 (Cochrane Collaboration) [24] by evaluating the following five critical domains: (1) random sequence generation, (2) allocation concealment, (3) blinding procedures, (4) completeness of outcome data, and (5) selective reporting, with additional consideration of other potential biases including small sample size effects and baseline characteristic imbalances. The methodological quality of cohort studies was evaluated by the Newcastle–Ottawa Scale [25], a semi-quantitative assessment tool structured across the following three domains: (1) participant selection (four items), (2) comparability of study groups (one item), and (3) outcome/exposure assessment (three items). The scoring system assigns one point for adequately addressed criteria in the selection and outcome/exposure domains, whereas the comparability domain allows up to two points based on the degree of bias control, with a zero point indicating insufficient methodological rigor in any domain. The methodological quality of cross-sectional studies was assessed with reference to the Joanna Briggs Institute Critical Appraisal Checklist for Analytical Cross-Sectional Studies [26], an eight-item instrument with four response options per criterion: "Yes" (criterion satisfied), "No" (criterion unmet), "Unclear" (insufficient reporting to determine compliance), and "Not Applicable" (criterion irrelevant to study design).

### Data synthesis

We were unable to conduct a meta-analysis of all included study exposure-endings, considering the lack of exposure-endings correlation studies in each group and the heterogeneity of effect sizes. Therefore, for pairs in which more than two studies reported the same exposure-outcome, we performed a meta-analysis. For those with inadequate numbers of studies, we only described the important characteristics and findings.

### Statistic analysis

Review Manager 5.4 and Stata 15 were used to analyze the data. Effect sizes were presented using odd ratios (OR) values and 95% confidence intervals (CI). Meta-analysis was performed using a random-effects inverse-variance model, and heterogeneity variance was estimated using the DerSimonian–Laird method. Forest plots were employed to determine the characteristics of meta-analysis. Between-study heterogeneity was analyzed using the I^2^ test. Less than 10 studies were selected for meta-analysis, and no funnel plot analysis for publication bias was conducted [27].

## Results

**Fig. 1:**
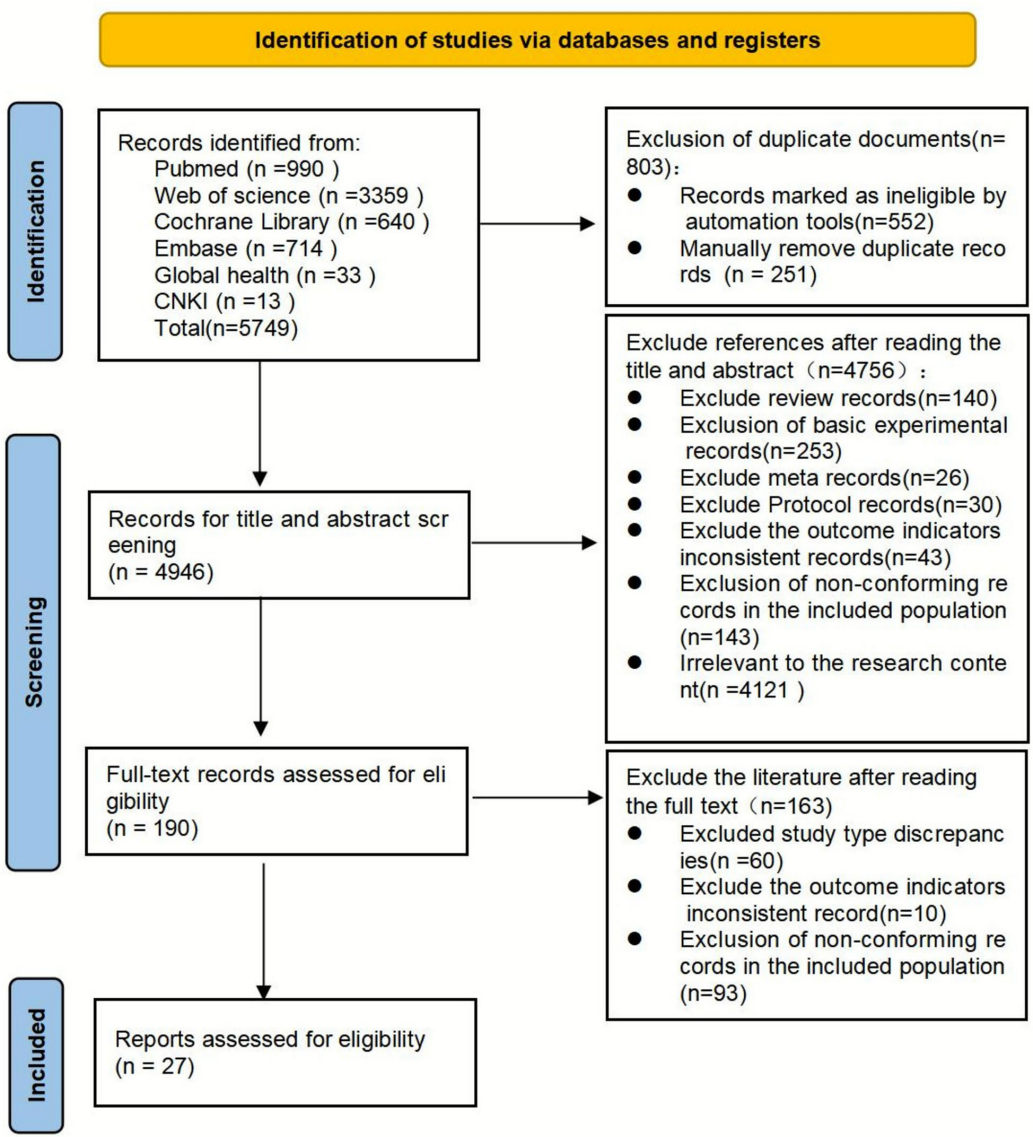
PRISMA flow diagram

The retrieval process was documented using the PRISMA flow diagram (Fig. 1). In total, 5,749 records were initially identified. After removing 803 duplicates, 4,946 articles remained. After screening the titles and abstracts, 4756 studies were excluded, leaving 190 for full-text review. Upon detailed examination, 163 articles were excluded for not meeting the inclusion criteria, resulting in 27 studies being included in this review.

### Characteristics of the included studies

This systematic review encompassed 27 publications from 2012 to 2024, comprising 14 longitudinal studies, 12 cross-sectional studies, and one RCT. The geographical distribution of studies varied by design. Among the longitudinal studies (*n =* 14), the majority originated from China (29%) [28–31] and the United States (57%) [32–39], with one study each from Spain [40] and Belgium [41]. The cross-sectional studies (*n =* 12) displayed a similar pattern, with 42% conducted in China [42–46] and 33% in the United States [47–50], along with single studies from the United Kingdom [51], Norway [52], and Malaysia [53]. The only RCT was conducted by a Chinese research team [54]. All studies targeted populations aged ≥ 60 years, with sample sizes ranging widely from 24 to 63,009,173 participants (Table 2)*.*

**Table 2:** Descriptive characteristics of included studies

Title	Author	Country	Publication	Year	Study Design	Sample Size	Age	Sexuality	Study Period	Type of Greenspace	The distance of green space	NDVI score	Outcome index	Main Results
Association between residential greenness and depression symptoms in Chinese community-dwelling older adults	Pengfei Wang et al.	China	Environmental Research	2024	Cross-sectional	7512	≥ 60	Male:3481 Female:4031	2019–2021	Residential greening	250、1000	0.34	mental illness	The higher the residential greenness (measured by NDVI and EVI), the lower the incidence of depressive symptoms. (*OR*: 0.045; 95% CI: 0.015–0.133)
The association of access to green space with low mental distress and general health in older adults: a cross-sectional study	Heidi Lyshol et al.	norway	BMC Geriatrics	2024	Cross-sectional	2068	≥ 65	Male:1065 Female:1003	2015–2016	Green space accessibility	Not described	Not described	mental illness	The elderly with self-reported ' easy access to green space ' were significantly more likely to have low psychological distress (*OR* = 3.85,95% CI 2.04–6.02)
Exposure to green spaces, cardiovascular risk biomarkers and incident cardiovascular disease in older adults: The Seniors-Enrica II cohort	Cara Scheer et al.	Spain	Environment International	2024	Longitudinal	2114	≥ 65	Male:951 Female:1162	2015–2022	The surrounding greenness of Normalized Difference Vegetation Index (NDVI)	250, 500, 750 and 1000	Not described	circulatory disease	In the 500 m, 750 m, and 1000 m buffer zones, the increased IQR of green space exposure was associated with a decreased risk of cardiovascular disease, with hazard ratios (*HR*) of 0.62 (95% CI: 0.38–1.00), 0.63 (0.38–1.03), and 0.57 (0.33–0.99), respectively The association in the 250 m buffer was not statistically significant (*HR *= 1.02, 95% CI: 0.60–1.74)
Longitudinal Impacts of High Versus Low Greenness on Cardiovascular Disease Conditions	Scott C. Brown et al.	USA	Journal of the American Heart Association	2024	Longitudinal	229,034	≥ 65	Male:96,283 Female:132,751	2011–2016	The surrounding greenness of Normalized Difference Vegetation Index (NDVI)	Not described	2011: 0.35 2016: 0.43	circulatory disease	Residents in the High-High area had a significantly reduced risk of new cardiovascular diseases (including myocardial infarction, atrial fibrillation, heart failure, etc.) by 9% within 5 years (adjusted odds ratio *OR* = 0.91,95% CI: 0.84–0.99, p= 0.021)
Longitudinal Impacts of Precision Greenness on Alzheimer's Disease	S.C. Brown et al.	USA	Journal of Prevention of Alzheimer's Disease (J Prev Alz Dis)	2024	Longitudinal	230,738	≥ 65	Male:96,913 Female:133,825	2011–2016	The surrounding greenness of Normalized Difference Vegetation Index (NDVI)	100–500 range	2011: 0.35 2016: 0.43	neurological disease	The incidence of AD decreased by 16% (*OR* = 0.84,95% CI: 0.76–0.94, p = 0.0014) in the elderly living in continuous high green areas
Residential greenspace and major depression among older adults living in urban and suburban areas with different climates across the United States	Alan J. Fossa et al.	USA	Environmental Research	2024	Longitudinal	21,611	≥ 65	Male:9725 Female:11,886	2008–2016	The surrounding greenness of Normalized Difference Vegetation Index (NDVI)	1000	0.78	mental illness	Residential green space may reduce the risk of major depression in the elderly through a climate-dependent mechanism (*PR* = 0.91,95% CI: 0.84–0.98)
Utilizing regression model to characterize the impact of urban green space features on the subjective well-being of older adults	Tianrong Xu et al.	Malaysia	Heliyon	2024	Cross-sectional	536	≥ 60	Male:275 Female:261	2022	The proximity of various green spaces	Not described	Not described	life satisfaction	Spatial, green and gray characteristics were significantly correlated with the overall satisfaction and subjective well-being of the elderly (*p <* 0.05)
When healthy aging meets Vitamin G: Assessing the associations between green space and heart health in older adults using street view and electrocardiography	Ruoyu Wang et al.	China	Landscape and Urban Planning	2024	Cross-sectional	3942	≥ 60	Male:2,231 Female:1,711	2010–2012	The surrounding greenness of Normalized Difference Vegetation Index (NDVI)	1000	0.299	circulatory disease	Street landscape grassland (rather than trees or remote sensing vegetation index) has a protective effect on the heart health of the elderly (*OR* = 0.876,95% CI: 0.816–0.940)
Association of residential greenness with the risk of metabolic syndrome in Chinese older adults: a longitudinal cohort study	P. Ke et al.	China	Journal of Endocrinological Investigation	2023	Longitudinal	49,893	≥ 65	Male:21,768 Female:28,125	2018–2020	Residential greening	250, 500 and 1250	250 m: 0.33 500 m: 0.33 1250 m: 0.35	metabolic disorders	The nearer the greenness (such as 250 m), the stronger the protective effect (trend test were significant P-trend < 0.01)
Neighborhood greenspace and cognition: The cardiovascular health study	Sara L. Godina et al.	USA	Health & Place	2023	Longitudinal	2141	The average age was 75.3 years	Male:925 Female:1216	1998–1999	Forest coverage (canopy coverage), green space type diversity	5000	Not described	cognitive disorder	The highest quartile (Q4) of forest coverage and green space diversity were associated with a decreased risk of MCI (*OR* = 0.54,95% CI: 0.29–0.98) (*HR* = 0.70,95% CI: 0.50–0.99)
Urban overall and visible greenness and diabetes among older adults in China	Kejia Hu et al.	China	Landscape and Urban Planning	2023	Cross-sectional	3924	≥ 65	Male:1,856 Female:2,068	2017–2018	The surrounding greenness of Normalized Difference Vegetation Index (NDVI)	500	0.18	metabolic disorders	The risk of diabetes in the highest quartile of NDVI (Q4) was 52% lower than that in the lowest quartile (Q1) (*OR*: 0.48,95% CI: 0.37–0.62)
Association of residential greenness with geriatric depression among the elderly covered by long-term care insurance in Shanghai	Wenjia Peng et al.	China	Environmental Science and Pollution Research	2022	Cross-sectional	1066	≥ 60	Male:439 Female:627	2018	NDVI and Soil Adjusted Vegetation Index (SAVI)	100, 300 and 500	100 m: 0.286 300 m: 0.285 500 m: 0.296	mental illness	The prevalence of geriatric depression decreased by 11.9% (*PR*: 0.881, 95% CI: 0.795–0.977) for each quartile increase in NDVI (Normalized Difference Vegetation Index) (IQR)
Associations between residential greenness and blood lipids in Chinese elderly population	J. Xu et al.	China	Journal of Endocrinological Investigation	2022	Longitudinal	34,563	≥ 65	Male:15,450 Female:19,113	2018–2020	Residential greening	250, 500 and 1250	250 m: 0.319 500 m: 0.323 1250 m: 0.340	metabolic disorders	The greening degree of residential areas was significantly correlated with the improvement of blood lipid metabolism in the elderly, especially the close greening (NDVI250-m) (p nonlinear < 0.05)
The influence of green space on the subjective well-being of the elderly in cold regions: A case study of Changchun City	Bingbing Han et al.	China	Landscape Architecture	2022	Cross-sectional	513	≥ 60	Male:278 Female:235	2018–2019	The proximity of various green spaces	1000	Not described	life satisfaction	Green space accessibility (such as nearest park distance and length of stay) had the most significant impact on life satisfaction (*p<* 0.01)
Associations of parks, greenness, and blue space with cardiovascular and respiratory disease hospitalization in the US Medicare cohort	Jochem O. Klompmaker et al.	USA	Environmental Pollution	2022	Longitudinal	63,009,173	≥ 65	Male:28,291,119 Female:34,718,054	2000–2016	Park coverage, NDVI (green index)	Not described	0.52	circulatory disease	In the overall population, for every 0.27 (IQR) increase in NDVI, the risk of cardiovascular disease (CVD) hospitalization decreased by 3% (*HR*: 0.97, 95% CI: 0.96–0.97)
The effects of greenness exposure on hypertension incidence among Chinese oldest-old: a prospective cohort study	Zhou Wensu et al.	China	Environmental Health	2022	Longitudinal	5253	≥ 80	Male:2052 Female:3201	2008–2018	The surrounding greenness of Normalized Difference Vegetation Index (NDVI)	500	0.46	circulatory disease	The potential protective effect of green space on hypertension was confirmed in the super-aged population in China (*HR* = 0.60; 95% CI: 0.53–0.70)
Long-term exposure to residential greenness and neurodegenerative disease mortality among older adults: a 13-year follow-up cohort study	Lucía Rodriguez-Loureiro et al.	Belgium	Environmental Health	2022	Longitudinal	1,134,502	≥ 60	Male:490,105 Female:644,397	2001–2014	The surrounding greenness of Normalized Difference Vegetation Index (NDVI)	300, 500 and 1000	0.61	neurological disease	For every quartile increase in the greening degree of the residential area (IQR = 0.22), the premature mortality rate caused by all neurodegenerative diseases decreased by 4–5%
Neighborhood greenspace exposure as a protective factor in dementia risk among U.S. adults 75 years or older: a cohort study	Erik D. Slawsky et al.	USA	Environmental Health	2022	Longitudinal	3047	≥ 75	Male:1636 Female:1411	2000–2008	Composite index (NDVI, park coverage, nearest park distance)	2000	0.57	neurological disease	High/medium green space in residential areas was associated with a reduced risk of all-cause dementia in the elderly (*HR* = 0.72,95% CI: 0.55–0.95)
Associations between neighborhood greenspace and brain imaging measures in non-demented older adults: the Cardiovascular Health Study	Lilah M. Besser et al.	USA	Social Psychiatry and Psychiatric Epidemiology	2021	Longitudinal	1125	≥ 65	Male:478 Female:647	2020	Neighborhood greening	1000、5000	Not described	cognitive disorder	A higher proportion of community green space was marginally significantly associated with lower ventricular enlargement (estimated value: − 0.30; 95% CI: −0.61, 0.00; p = 0.052)
Relationship of Neighborhood Greenness to Alzheimer's Disease and Non-Alzheimer's Dementia Among 249,405 U.S. Medicare Beneficiaries	William W. Aitken et al.	USA	Journal of Alzheimer’s Disease	2021	Cross-sectional	249,405	≥ 65	Male:104,750 Female:144,655	2010–2011	The surrounding greenness of Normalized Difference Vegetation Index (NDVI)	Not described	−0.02	neurological disease	Community greenness was significantly associated with reduced risk of AD and ADRD (*OR* = 0.94,95% CI: 0.88–1.00) (*OR* = 0.93,95% CI: 0.88–0.99)
APOE ε4 Modifies Effect of Residential Greenness on Cognitive Function among Older Adults: A Longitudinal Analysis in China	Anna Zhu et al.	China	Scientific Reports	2020	Longitudinal	6994	≥ 65	Male:3400 Female:3594	2000–2014	Residential greening	500	0.41	cognitive disorder	APOE ε4 genotype significantly increased the risk of cognitive impairment and weakened the protective effect of residential greenness (*OR*: 0.83, 95% CI: 0.72–0.95), which was more pronounced in younger elderly (65–79 years old) ((*OR*: 0.76))
Neighbourhood greenness and depression among older adults	Tatiana Perrino et al.	USA	The British Journal of Psychiatry	2019	Longitudinal	249,405	≥ 65	Male:104,750 Female:144,655	2010–2011	community greening	Not described	−0.02	mental illness	There was a significant negative correlation between the community greening degree (NDVI) and the diagnosis rate of depression in the elderly population. (*OR* = 0.91; 95% CI 0.86–0.96)
Relationship of Neighborhood Greenness to Heart Disease in 249 405 US Medicare Beneficiaries	Kefeng Wang et al.	USA	Journal of the American Heart Association	2019	Cross-sectional	249,405	≥ 65	Male:103,927 Female:145,478	2010–2011	The surrounding greenness of Normalized Difference Vegetation Index (NDVI)	Not described	−0.02	circulatory disease	Increased community greenness was associated with a decreased risk of heart disease in the elderly (*OR* = 0.81,95% CI: 0.78–0.84)
Health Disparities in the Relationship of Neighborhood Greenness to Mental Health Outcomes in 249,405 U.S. Medicare Beneficiaries	Scott C. Brown et al.	USA	International Journal of Environmental Research and Public Health	2018	Cross-sectional	249,405	≥ 65	Male:103,927 Female:145,478	2010–2011	Vegetation coverage assessed using the normalized difference vegetation index (NDVI)	Not described	−0.02	Alzheimer 's disease and depression	Greenness was negatively correlated with the risk of Alzheimer 's disease and depression in the elderly population, and the improvement of greenness on depression was more significant in low-income communities. (*OR* = 0.790, *p <* 0.0001)
Neighborhood Greenness and Chronic Health Conditions in Medicare Beneficiaries	Scott C. Brown et al.	USA	American Journal of Preventive Medicine	2016	Cross-sectional	249,405	≥ 65	Male:103,927 Female:145,478	2010–2014	community greening	Not described	−0.02	chronic disease	Community greening (NDVI) was significantly associated with reduced risk of chronic diseases (diabetes, hypertension, hyperlipidemia) in the elderly (*OR* = 0.918,95% CI: 0.900–0.937) (*OR *= 0.927,95% CI: 0.911–0.943) (*OR* = 0.941,95% CI: 0.924–0.959)
Older people, the natural environment and common mental disorders: cross-sectional results from the Cognitive Function and Ageing Study	Yu-Tzu Wu et al.	UK	BMJ Open	2015	Cross-sectional	2424	≥ 74	Male:970 Female:1454	1991–2001	Percentage of green space and private gardens	Not described	Not described	mental illness	Exposure to high natural environment in the community was significantly associated with a reduced risk of mental disorders in the elderly (*OR* = 0.55,95% CI 0.35–0.84)
Therapeutic effect of forest bathing on human hypertension in the elderly	Gen-Xiang Mao et al.	China	Journal of Cardiology	2012	RCT	24	60–75	Not described	2011	forest	Not described	Not described	circulatory disease	Short-term forest bath can significantly reduce blood pressure in elderly hypertensive patients, inhibit the renin-angiotensin system and inflammatory response (*p <* 0.05)

### Risk of bias

**Fig. 2:**
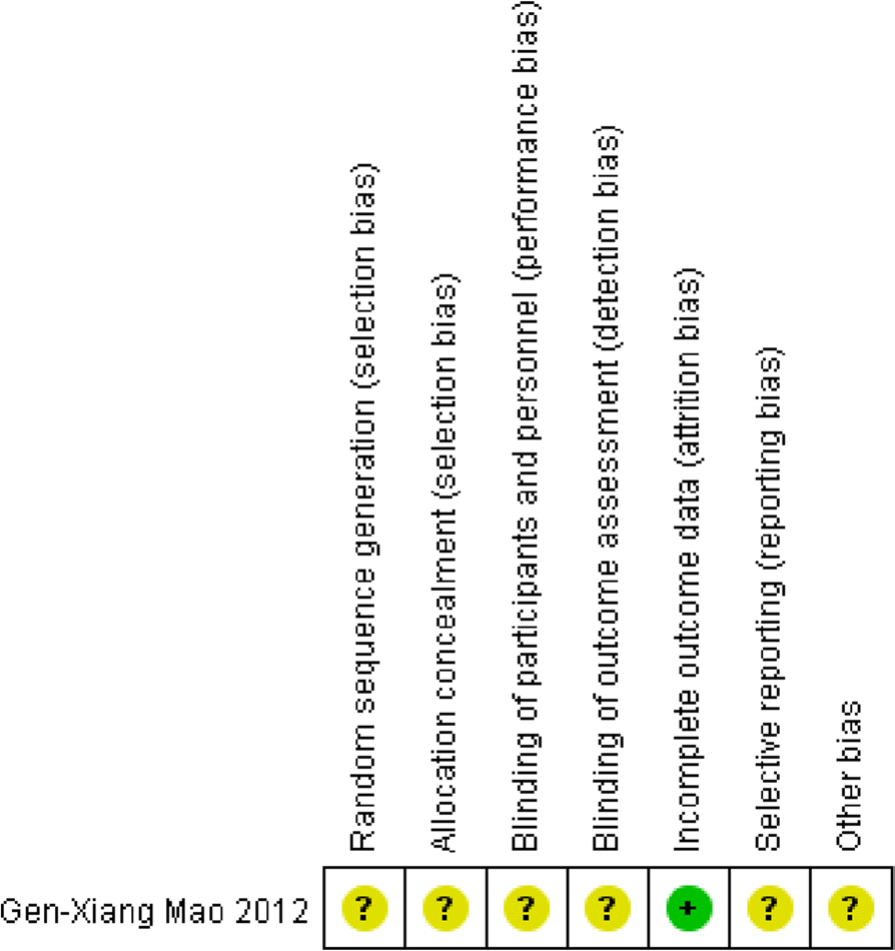
Risk of bias for randomized controlled trial

Quality assessment of the 14 longitudinal studies indicated that all achieved high-quality scores (range: 6–8). However, closer examination revealed certain potential limitations. Four studies had inconsistent baseline data [28, 29, 38, 41], and three studies reported relatively short follow-up periods [28, 29, 32]. Notably, none of the included longitudinal studies reported dropout rates, which could influence the accuracy of the findings. Among the 12 cross-sectional studies, most were rated as having medium-to-high quality. Two studies had lower quality scores due to unclear inclusion and exclusion criteria [46, 53] and inadequate control of confounding factors. Overall, most cross-sectional studies demonstrated appropriate sample selection, valid measurement methods, and sound data analysis. Nevertheless, some deficiencies were identified, including limited control of confounders, insufficient reliability and validity of measurement tools for outcome indicators, and incomplete baseline data. For the single RCT, the risk of bias assessment indicated that most bias-related factors could not be clearly determined [54]. The detailed quality assessments of the included studies are presented in Tables 3, 4, and Fig. 2.

**Table 3:** Detailed Newcastle–Ottawa Scale of each included Longitudinal study

Authors	Selection												Comparability			Outcome									Total score	Quality
	**Representativeness of the exposed cohort**			**Selection of the non exposed cohort**			**Ascertainment of exposure**			**Demonstration that outcome of interest was not present at start of study**			**Comparability of cohorts on the basis of the design or analysis**			**Assessment of outcome**			**Was follow-up long enough for outcomes to occur**			**Adequacy of follow up of cohorts**				
	2	1	0	2	1	0	2	1	0	2	1	0	2	1	0	2	1	0	2	1	0	2	1	0		
Anna Zhu et al.	/	√		/	√		/	√		/	√		√			/		√	/	√		/		√	7	Good
P. Ke et al.	/	√		/	√		/	√		/		√	√			/	√		/		√	/		√	6	Good
Lilah M. Besser et al.	/	√		/	√		/	√		/	√		√			/	√		/		√	/		√	7	Good
J. Xu et al.	/	√		/	√		/	√		/		√	√			/	√		/		√	/		√	6	Good
Jochem O. Klompmaker et al.	/	√		/	√		/	√		/	√		√			/	√		/	√		/		√	8	Good
Zhou Wensu et al.	/	√		/	√		/	√		/	√		√			/	√		/	√		/		√	8	Good
Cara Scheer et al.	/	√		/	√		/	√		/	√		√			/	√		/	√		/		√	8	Good
Scott C. Brown et al.	/	√		/	√		/	√		/	√		√			/		√	/	√		/		√	7	Good
Scott C. Brown et al.	/	√		/	√		/	√		/	√		√			/		√	/	√		/		√	7	Good
Lucía Rodriguez-Loureiro et al.	/	√		/	√		/	√		/		√	√			/	√		/	√		/		√	7	Good
Sara L. Godina et al.	/	√		/	√		/	√		/	√		√			/	√		/	√		/		√	8	Good
Erik D. Slawsky et al.	/	√		/	√		/	√		/	√		√			/	√		/	√		/		√	8	Good
Alan J. Fossa et al.	/	√		/	√		/	√		/	√		√			/		√	/	√		/		√	7	Good
Tatiana Perrino et al.	/	√		/	√		/	√		/		√	√			/	√		/	√		/		√	7	Good

**Table 4:** Detailed JBI analytical cross-sectional study quality assessment checklist for each included cross-sectional study

Authors	Were the criteria for inclusion in the sample clearly defined?				Were the study subjects and the setting described in detail?				Was the exposure measured in a valid and reliable way?				Were objective, standard criteria used for measurement of the condition?				Were confounding factors identified?				Were strategies to deal with confounding factors stated?				Were the outcomes measured in a valid and reliable way?				Was appropriate statistical analysis used?			
	**Yes**	**No**	**Unclear**	**Not Applicable**	**Yes**	**No**	**Unclear**	**Not Applicable**	**Yes**	**No**	**Unclear**	**Not Applicable**	**Yes**	**No**	**Unclear**	**Not Applicable**	**Yes**	**No**	**Unclear**	**Not Applicable**	**Yes**	**No**	**Unclear**	**Not Applicable**	**Yes**	**No**	**Unclear**	**Not Applicable**	**Yes**	**No**	**Unclear**	**Not Applicable**
Pengfei Wang et al.	√				√				√				√				√				√				√				√			
Heidi Lyshol et al.	√				√				√				√				√				√				√				√			
Wenjia Peng et al.	√				√				√				√				√				√				√				√			
Scott C. Brown et al.	√				√				√				√				√				√				√				√			
Scott C. Brown et al.	√				√				√						√		√				√				√				√			
Yu-Tzu Wu et al.	√				√				√						√		√				√				√				√			
William W. Aitken et al.	√				√				√					√			√				√				√				√			
Kefeng Wang et al.	√				√				√					√			√				√						√		√			
Kejia Hu et al.		√			√				√					√			√				√						√		√			
Tianrong Xu et al.	√				√					√					√			√				√					√		√			
Ruoyu Wang et al.		√			√				√					√			√				√				√				√			
Bingbing Hanet al	√				√				√						√			√				√			√				√

### Green space attributes

The green space attributes assessed in this systematic review fall into three main categories: green coverage level, accessibility, and ecological structure characteristics. Green coverage metrics included direct indicators such as residential greening [28, 29, 31, 42], community greening [38, 47, 48], neighborhood landscaping [32], park cover [36, 37], and forest/canopy coverage [35, 54]. They also encompassed measures of surrounding green cover derived from NDVI, quantified through remote sensing technology [30, 33, 34, 36, 37, 39–42, 44, 45, 49, 50]. Accessibility indicators emphasized the proximity and availability of green resources such as parks, green spaces, and forests, including the distance to the nearest park and the density of green spaces within a walkable range [46, 52, 53]. At the ecological structure level, the focus was on composite attributes such as the diversity of green space types and the proportion of private gardens [37, 51]. Across the included studies, most exposure to green space measurements were based on the participants’ place of residence, with the most common buffer zones ranging from 250 m to 1,000 m.

### Health outcomes

Our findings indicated that the included health outcomes encompassed circulatory system diseases, metabolic diseases, nervous system disorders, cognitive impairments, mental illnesses, and life satisfaction [28–54].

Seven studies examined CVDs. Two of these reported associations between exposure to green space and variations in blood pressure among older adults [30, 54]. Another two investigated the effects of exposure to green space on heart health specifically [45, 50]. The remaining studies evaluated the association between exposure to green space and CVD from a broader perspective [33, 36, 40].

Three studies focused on metabolic diseases, investigating associations with metabolic disorders in general [28], dyslipidemia [29], and diabetes [44].

Seven studies addressed nervous system diseases. The majority examined the effects of exposure to green space on Alzheimer’s disease (AD) [34, 37, 41, 49] and cognitive impairment [31, 32, 35]. Two studies also explored the impact of exposure to green space on neurodegenerative diseases more broadly [37, 41].

In total, six mental illness studies were included. Four studies focused on geriatric depression [38, 39, 42, 43], one examined both geriatric depression and geriatric anxiety [51], and one assessed overall mental health in older adults [52].

Two studies evaluated the effects of exposure to green space on life satisfaction [46, 53]. Additionally, one study analyzed the relationship between exposure to green space and the prevalence of common chronic diseases in older adults [48], while another simultaneously examined associations with both AD and depression [47].

### Link between green space and health outcomes

#### Green space and circulatory system diseases

Seven studies, including four cohort studies, two cross-sectional investigations, and one RCT, examined the relationship between exposure to green space and circulatory diseases such as hypertension, heart disease, and CVD. Together, these studies included 6,349,894 participants, with sample sizes ranging from 24 to 63,009,173 individuals. A meta-analysis was not performed because of substantial variation in the effect size metrics used to report clinical outcomes. Associations between exposure to green space and health outcomes were measured using indicators such as forest and park coverage, as well as mean NDVI values representing surrounding vegetation. Two studies categorized exposure into low, medium, and high levels [33, 50], while others calculated NDVI values within buffer zones of 250 m, 500 m, 750 m, and 1,000 m around participants’ homes to determine green space density.

Across the seven studies, the findings on the cardiovascular benefits of green space for older adults were not entirely consistent [30, 33, 36, 40, 45, 50, 54], although a considerable amount of evidence supports a protective effect. In studies on hypertension, one investigation reported a nonlinear, U-shaped association between exposure to green space and the risk of hypertension. The protective effect was especially notable in areas with high green coverage, where each 0.1-unit increase in NDVI was associated with a 40% reduction in hypertension risk [30]. Another study found that exposure to green space also contributed to the management of hypertension in older adults [54]. In an RCT, elderly residents exposed to a forest environment experienced significant decreases in diastolic blood pressure, low-frequency heart rate variability, and high-sensitivity C-reactive protein, as well as significant increases in oxygen saturation and high-frequency heart rate variability [55]. Two studies showed that high exposure to green space was associated with a reduced risk of CVD [33, 36]. Living in greener communities was associated with a 25% lower risk of acute myocardial infarction, a 20 percent lower risk of ischemic heart disease, and a 16% lower risk of heart failure [45, 50]. Ruoyu Wang et al. also found that a greater proportion of visible green space in the community was associated with better cardiovascular health in older adults [45]. Three studies reported no statistically significant association. A cohort study in Spain found no significant link between green space within a 250-m buffer and CVD, although green space within a 500–1,000 m buffer was associated with lower levels of CVD biomarkers, including N-terminal pro–B-type natriuretic peptide, high-sensitivity troponin, and interleukin-6 [40]. In a study of the oldest-old population in China, no significant association was found between low green cover and hypertension risk [30]. Another Chinese study found no significant relationship between the presence of street trees and cardiovascular health in older adults.

#### Green space and metabolic diseases

Three studies, comprising two cohort studies and one cross-sectional study, examined the relationship between exposure to green space and MetS in a total of 88,380 older adults. These studies investigated different health outcomes, including diabetes mellitus, dyslipidemia, and MetS. All three studies used NDVI to quantify exposure to green space, although they differed in the spatial scales applied. One study calculated NDVI within a single 500-m residential buffer zone [44], while the other two used multiple buffer zones centered on participants’ geocoded residential addresses. Across the three studies, a higher level of exposure to green space was consistently associated with better metabolic health [28, 29, 44]. One study reported that for every 0.1-unit increase in NDVI, the odds of being free from chronic diseases such as diabetes, hypertension, and hyperlipidemia increased by 7% in the overall sample [48]. Another study found that greater residential greenery was associated with a lower risk of MetS [28]. Interestingly, one investigation showed that while higher levels of greenery were generally protective, the strength of the association with blood lipid levels diminished as green space increased [29]. Additionally, overall green coverage was associated with a reduced risk of diabetes, but the proportion of greenery visible in daily life was not significantly associated with diabetes risk [44]. A meta-analysis was not performed because of differences in outcome measures and effect size reporting across studies.

#### Green space and nervous system diseases

**Fig. 3:**
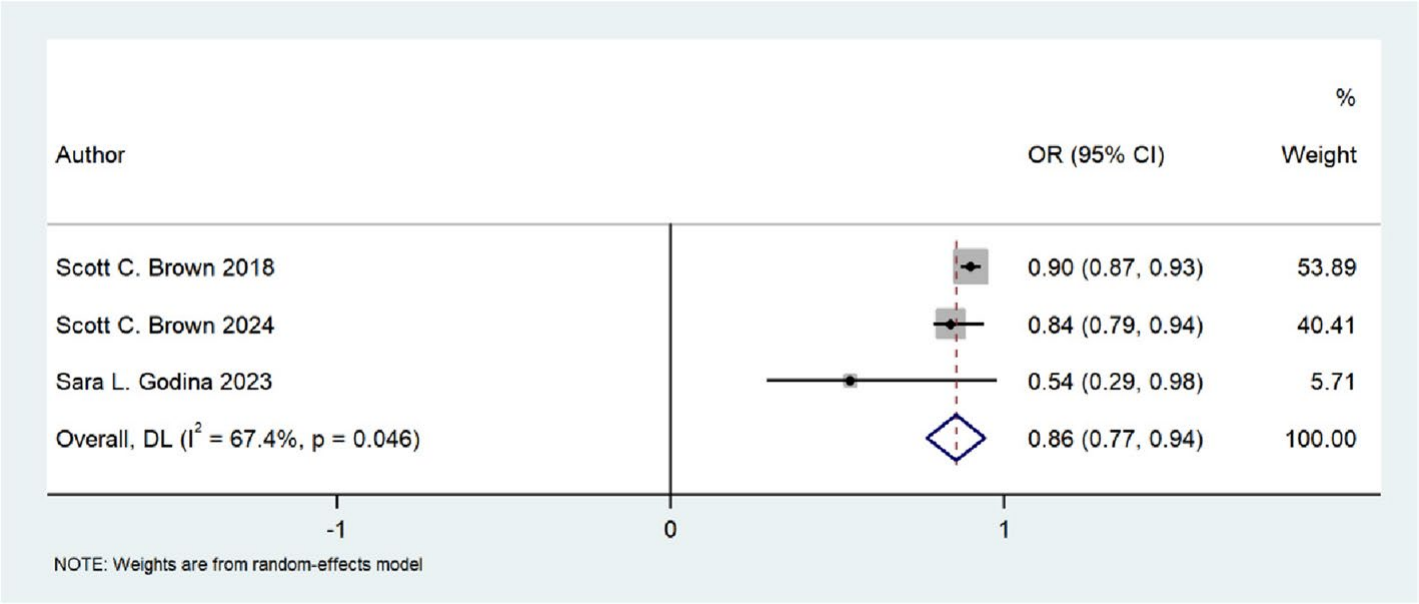
Forest plot of the effect of green space exposure on Alzheimer's disease

Seven studies, including four cohort and three cross-sectional designs, investigated the association between exposure to green space and neurological disorders. The combined sample size across these studies was 1,877,357 participants, with individual sample sizes ranging from 1,125 to 1,134,502. Most studies reported that higher levels of residential greenery were associated with a lower risk of AD or dementia, as well as better cognitive function [31, 32, 34, 35, 37, 41, 49]. Two cross-sectional studies conducted in the United States found that continuous high exposure to green space was associated with a 16%–20% reduction in AD incidence among older adults [34, 49]. Another study examining both AD and depression found that for every 0.1-unit increase in NDVI, the risk of AD decreased by 10% [47]. A Belgian study reported that for every one interquartile range increase in greenery, total mortality from neurodegenerative diseases decreased by 4%–5% [41]. Research by Brown et al. showed that younger older adults (age: 65–74 years) living in greener communities had a reduced risk of developing AD and reported better overall health [34]. The study suggested that these protective effects were mediated through increased physical activity, reduced psychological stress, and stronger social support networks [34]. Among studies on cognitive impairment, one found that forest coverage was more strongly associated with dementia risk than other green space measures [35]. The APOEε4 gene, a major genetic risk factor for AD, may reduce the protective effects of green space on cognitive function, as reported in several studies [31, 32]. Because of variations in exposure measurements, outcome definitions, and available data, a meta-analysis was conducted for only three studies that reported on AD. The pooled analysis indicated a significant protective effect of exposure to green space on AD in older adults (OR: 0.856, 95% CI: 0.769–0.943), with moderate heterogeneity (I^2^ = 67.4%, p = 0.046) [34, 35, 47] (Fig. 3).

#### Green space and mental illness

**Fig. 4:**
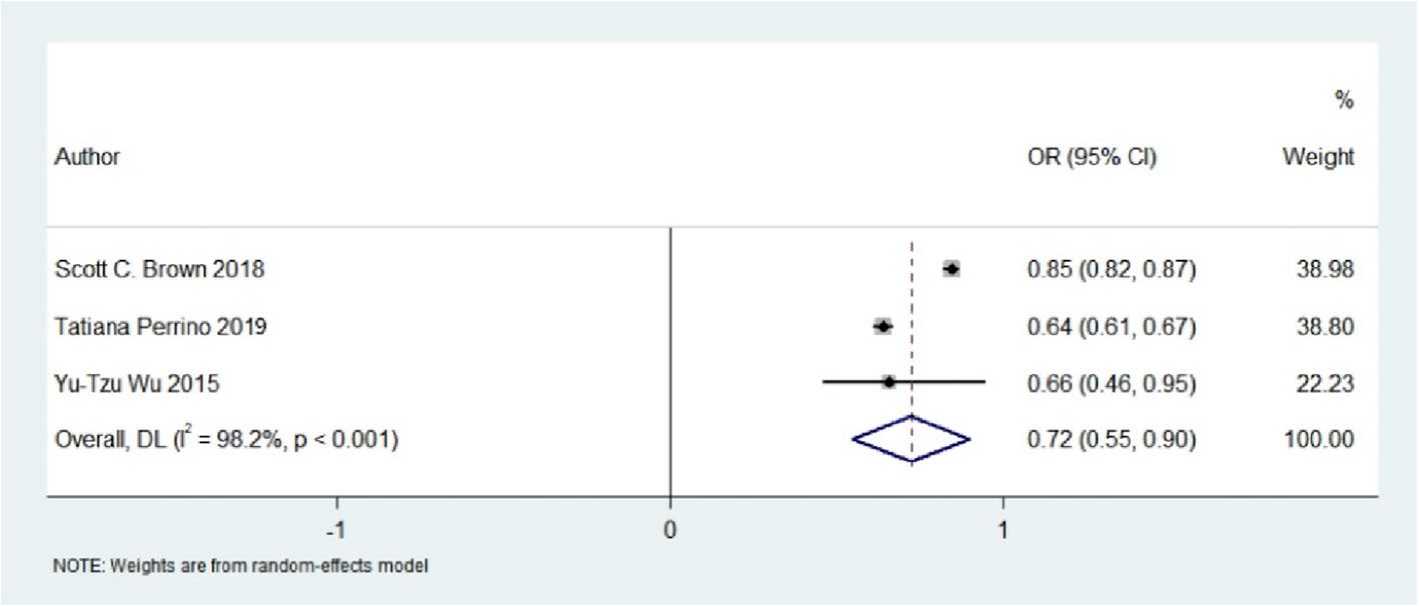
Forest plot of the effect of green space exposure on depression

Six observational studies examined the relationship between exposure to green space and geriatric mental health outcomes in a pooled sample of 284,086 participants, comprising five cross-sectional studies and one cohort study. Five investigations assessed composite mental health indices reflecting overall psychological status, while one study specifically focused on late-life depressive disorders [38, 39, 42, 43, 47, 52]. One study measured both depressive symptoms and anxiety disorders using validated clinical instruments [51]. Another study on AD and depression found that for every 0.1 increase in NDVI, the risk of depression decreased by 28% [47]. All included studies reported that exposure to green spaces was associated with a reduced risk of mental illnesses among older adults [38, 39, 42, 43, 47, 51, 52]. A Norwegian cohort study observed an inverse relationship between residential exposure to green space and psychological distress in older adults, with a stronger dose–response effect in those with better baseline physical health [52]. In contrast, studies from China found that the association between exposure to green space and depressive symptoms was mediated by physical activity [42, 43]. Research from Shanghai and the UK suggested that green communities reduce depression in older adults by fostering social cohesion [43, 51]. Another study reported a 15% reduction in depression risk for each 0.1-unit increase in NDVI [47]. Due to differences in exposure measures and reported outcomes, as well as limited data, a meta-analysis was conducted for only three studies on depression. The pooled results indicated a protective effect of exposure to green space on depression in older adults (OR: 0.724, 95% CI: 0.549–0.900), with high heterogeneity (I^2^ = 99.5%, p = 0.000) [38, 47, 51] (Fig. 4).

#### Green space and life satisfaction

Two studies examined the impact of exposure to green space on life satisfaction in older adults [46, 53], including 1,049 participants. A meta-analysis was not conducted due to heterogeneity in effect size metrics. Both studies found that green spaces positively influenced life satisfaction by providing opportunities for outdoor activity, fostering social interaction, and offering aesthetic enjoyment [56]. Enhanced environmental quality from green spaces was shown to have both direct and indirect effects on residents’ life satisfaction. One study highlighted a positive association between green viewing rate and park accessibility with life satisfaction in older adults [46]. Another study found that green spaces improved life satisfaction primarily through enhanced environmental quality and a stronger sense of place among residents [53].

Although we have already described the heterogeneity of each outcome in the results section, some details still need to be elaborated on. These details are provided in Supplementary 1.

## Discussion

This systematic review identified 27 studies that examined the association between exposure to green space and health outcomes among older adults. The number of participants in these studies ranged from 24 to 63,009,173. Notably, only one study did not report gender distribution [54].

This review systematically analyzed and comprehensively evaluated the impact of exposure to green space on health indicators in older adults. The findings indicated that most evidence consistently supported the beneficial effects of exposure to green space on a range of chronic diseases in this population, with the notable exception of CVD findings. This conclusion regarding the broader range of chronic diseases aligns closely with results from broader population-based studies. However, in the CVD–related studies included in this review, exposure to green space demonstrated a nonlinear, dose–response association with cardiovascular risk in older adults. For instance, a study in Denmark reported no significant correlation between the number of green spaces within 500 m or 1,000 m and CVD risk, but found a protective effect within a 5,000 m radius [57]. The cardiovascular benefits of high exposure to green space may be attributed to the diversity of green space types, which enhance functionality and expand residents’ daily activity ranges, thereby promoting health. Existing evidence has firmly established the close link between exposure to green space and human health. Critically, while systematic reviews and meta-analyses have firmly established the beneficial links between green space exposure and various health outcomes (including CVD) in broader populations [58, 59], the evidence base specific to older adults—a rapidly growing demographic facing unique challenges—remains notably limited. Older adults often face multiple comorbidities. Their pathological, physiological, and psychosocial needs are far more complex than those of younger adults. Despite their high vulnerability and disease burden, there is a current lack of systematic evidence detailing how green space exposure specifically impacts common diseases, including CVD with its observed nonlinear pattern, within this elderly cohort. This review directly addresses this critical gap by synthesizing existing research through systematic review and meta-analysis, focusing on the older adult population and their specific health contexts.

Due to differences in exposure measures and outcome metrics across the included studies, we conducted meta-analyses for only two outcomes: depression and cognition. The results revealed a protective effect of exposure to green space on both depression and cognitive function in older adults. Specifically, a 0.1-unit increase in NDVI was associated with a reduced risk of depression [60]. Other studies have reported that exposure to green space exerts more pronounced mental health benefits in specific age groups (such as 18–24, < 30, and 31–50 years) [61, 62]. For example, pregnant women living within 100 m of green space had an 18%–23% lower incidence of depressive symptoms [63]. Mental health benefits of exposure to green space operate through three primary pathways: physical, psychological, and social. At the cellular level, mitochondria are the “energy factories” of cells, and oxidative stress can impair their function, leading to insufficient neuronal energy supply and triggering apoptosis, which increases depression risk [64, 65]. Exposure to green space mitigates these effects by reducing oxidative stress [66]. Furthermore, higher air pollutant levels are strongly associated with increased risks of depression and anxiety [67–69]. Exposure to green space helps improve mental health by reducing both air and noise pollution. In addition, individuals living in greener areas are more likely to engage in physical activity and participate in social interactions, reducing the likelihood of developing mental health disorders [70, 71]. Exposure to green space alleviates depression and anxiety by lowering stress levels, enhancing residents’ sense of well-being, and strengthening social cohesion [72].

Existing evidence shows a positive correlation between exposure to green space and lifelong markers of AD and related disorders, including overall brain health [73]. In childhood, high levels of exposure to green space have been strongly associated with better intellectual development, working memory, spatial orientation, and executive function [74, 75]. In adulthood and older age, greater exposure is associated with improved cognitive performance across multiple domains and brain regions, along with a lower risk of AD and other dementias [76]. A recent study found that cognitive scores declined by 0.01 points for every 1% increase in green space coverage [77]. Green spaces promote cardiovascular health by encouraging physical activity, which in turn helps reduce cognitive decline [78]. They also improve cognitive outcomes by lowering air pollution levels and trapping particulate matter [79], while further protecting brain health through stress reduction and increased social engagement [80, 81].

The health benefits of exposure to green space among older adults vary according to socioeconomic status, degree of aging, access levels, and environmental conditions. Studies report stronger benefits for those with low socioeconomic status (such as limited income and education) and for younger seniors aged 65–74 years [33, 38, 41, 45]. This may reflect their greater reliance on local community resources, with green spaces providing essential settings for recreation and social connection [38]. In physically capable younger seniors, regular use of green spaces for structured outdoor exercise has been associated with better cardiovascular function, improved mental well-being, and greater mobility, underscoring the value of age-targeted urban planning. Some research indicates that the protective effect of green space only becomes apparent beyond a certain exposure threshold. For example, the link between high exposure and reduced hypertension risk is especially clear at higher coverage levels [37]. Health impacts may also differ depending on the type and diversity of green space. While higher coverage has been associated with lower risks of CVD, dementia, diabetes, and hypertension, larger total green space area alone does not consistently predict better health outcomes [49, 82]. Regional variations in green space characteristics and environmental conditions are critical determinants of their associated health benefits. For example, in northern regions, trees undergo seasonal defoliation, and vegetation becomes sparse during winter, which may cause the health-promoting effects of green spaces to be less pronounced during this season [45].

In the studies included in this review, most measurement standards for assessing the accessibility of green spaces used NDVI to evaluate the level of exposure to green space. The buffer zone range was between 100 and 5,000 m [21]. Evidence suggests that exposure to green spaces within a smaller buffer zone (100–800 m) is more strongly associated with mental health, whereas exposure within a larger buffer zone (more than 800 m) tends to have a greater impact on physical activity [83, 84]. Maas et al. also found that a buffer zone within 1 km shows the strongest correlation with anxiety disorders and depression [85]. The likely explanation for this finding is that a small buffer zone (100–800 m) corresponds to the distance residents can typically cover within a 10-min walk [86]. Such proximity is closely tied to daily activities, neighborhood interactions, and community engagement, thereby exerting a direct influence on mental health and social connectedness. Conversely, larger buffer zones (> 800 m) may yield greater environmental health benefits. Extensive green areas have been shown to filter harmful substances, such as particulate matter, from the air while also offering more space for physical activity, which in turn improves a range of health outcomes.

This study indicates that exposure to green spaces can improve the overall health of older adults, partly by reducing environmental pollution. It also shows that green spaces exert a mediating influence on the health benefits experienced by older adults. For instance, green spaces can further enhance overall health by improving air quality. Vegetation and green areas can absorb harmful substances such as nitrogen oxides and particulate matter from the atmosphere, thereby improving residents’ overall health status [87, 88].

This systematic review also found that older adults’ overall health, social support, and physical activity play pivotal mediating roles in the link between green spaces and health. Research demonstrates that green spaces enhance residents’ sense of well-being and satisfaction, reduce psychological stress, and protect mental health by offering visually appealing surroundings [89]. Green areas provide open spaces where residents can engage in various forms of physical activity, encouraging more frequent exercise and thus promoting overall well-being [90]. This conclusion was also supported by our findings. Older adults can enjoy more comfortable walking conditions in areas with higher levels of exposure to green space, which stimulates their willingness to walk and promotes regular physical activity. In public open spaces such as parks and green areas, older adults are also more likely to interact frequently with neighbors, strengthen social bonds, and reduce feelings of loneliness.

The process of rapid urbanization has worsened the unequal distribution of public resources, with the issue of equitable green space allocation becoming especially urgent. Historically, the distribution of environmental amenities in cities has been uneven, with privileged residents in affluent neighborhoods enjoying greater access to parks and other green spaces [91].The theory of environmental justice emphasizes that urban public green space resources should be distributed equitably to ensure that all social groups, especially low-income populations, older adults, and immigrant communities, can equally benefit from the environmental advantages they provide [92, 93]. Compared with nonvulnerable groups, vulnerable groups tend to experience greater health gains from access to green spaces [94]. Our findings further indicate that areas with higher-quality green spaces have better exposure levels for older adults, resulting in more pronounced health benefits. Therefore, in future policy-making processes, it is recommended to revise urban planning standards to expand green areas within the living environments of low-income older adults and to enhance the quality of available green spaces.

Furthermore, biodiversity and habitat quality within urban green spaces can influence how people use and engage with these areas [95]. Higher biodiversity in green spaces has been associated with improved attention restoration, enhanced human well-being, and better mental health outcomes [96, 97]. However, in all the studies we reviewed, the evaluation of exposure to green space was limited to three dimensions—availability, accessibility, and visibility. None of the included studies assessed biodiversity. Therefore, we recommend that future research incorporate biodiversity evaluation and systematically examine the health-promoting mechanisms associated with different types of green areas.

The present study offers several strengths. First, it is a systematic review specifically examining the effects of green space on older adults. To our knowledge, no comparable study has been conducted. Second, we synthesized the available evidence and proposed a theoretical framework to guide subsequent investigations. Nonetheless, this study has certain limitations. Many of the included studies did not specify the type of vegetation, which prevented a targeted analysis of vegetation-specific health effects and limited the comprehensiveness of the findings. Additionally, inconsistent definitions and measurements of outcome indicators across studies meant that fewer indicators could be included in the meta-analysis. The substantial heterogeneity observed in the two pooled indicators was primarily attributable to variations in measurement tools and differences in population characteristics.

## Conclusions

This research synthesized the multidimensional health impacts of green environments on aging populations through a systematic review and critical appraisal of empirical studies examining exposure to green space and health outcomes in older adults. The evidence-based framework identifies strong associations between green space accessibility and quality with geriatric health indicators, providing a solid empirical basis for developing nature-based behavioral interventions. These findings present actionable guidance for a wide range of stakeholders, particularly urban planners and public health policymakers, to integrate green infrastructure design into strategies for creating age-friendly, health-enhancing communities.

## Supplementary Information

Supplementary Material 1.

Supplementary Material 2.

## Data Availability

Data is provided within the manuscript or supplementary information files.

## Abbreviations

AD: Alzheimer's disease
CVD: Cardiovascular disease
MetS: Metabolic Syndrome
NDVI: Normalized Difference Vegetation Index
PEOS: Population, Exposure, Outcome, and Study Design
PRISMA: Preferred Reporting Items for Systematic Reviews and Meta-Analysis
PROSPERO: Prospective Register of Systematic Reviews
