# Supplementary material for: Association between green space exposure and elderly health: a systematic review and meta-analysis
Source: BMC Public Health. 2026 Jan 8;26:491. doi: 10.1186/s12889-025-26137-y (PMC12882215; doi:10.1186/s12889-025-26137-y)
Supplement: Supplementary file 1 — Supplementary Material 1. [file 12889_2025_26137_MOESM1_ESM.docx]

**Supplementary 1. Heterogeneity Assessment for Studies Ineligible for Meta-Analysis**

**Green space and circulatory system diseases**

The seven included studies focused on the association between green space exposure and circulatory system diseases in the elderly.The heterogeneity was significant among the seven studies. The reason for the heterogeneity was that only two of the included studies reported hypertension^[30,54]^ and heart disease^[45,50]^ as outcome indicators respectively, which did not meet the minimum requirement of conducting at least three independent investigations on the same exposing-outcome pair for meta-analysis. Therefore, we are unable to conduct a meta-analysis. Two studies examined the impact of green space exposure on hypertension in older adults. The studies showed heterogeneity in terms of research design (one was a cohort study and the other was a RCT), population characteristics (one study examined a population aged 60–75 years and the other examined a population aged ≥80 years), and exposure indicators (one study used the NDVI as a quantitative indicator of green space exposure, while the other assessed exposure based on the objective environment in the forest). Such indicators included higher negative oxygen ion concentration, more suitable temperature, higher humidity, lower noise, and a more comfortable climate. The studies also differed in their statistical methods. One study used heart rate as an indicator of the effect, while the other study used mean difference. Two studies on the impact of green space exposure on heart disease in older adults showed heterogeneity in population characteristics. The age structure of the study populations differed (one study included participants aged ≥60 years and the other included participants aged ≥65 years), as did the racial composition of the study populations (one study included participants from China and the other included participants from the United States). There was also heterogeneity in the population characteristics (variations in the ethnic groups studied: two studies were conducted in the United States and one was conducted in Spain) and in the statistical methods (varying effect sizes: two studies used HR and one used OR) in the three cardiovascular disease studies. Therefore, based on these findings, we could not conduct a meta-analysis of the seven studies on circulatory system diseases in older adults.

**Green space and metabolic diseases**

Due to the fact that the three included studies focused on different metabolic diseases (diabetes^[44]^, dyslipidemia^[29]^ and metabolic syndrome^[28]^ respectively). The diseases were too heterogeneous to be combined into a single meta-analysis. Meanwhile, there was also heterogeneity in the study designs (two cohort studies and one cross-sectional study) and statistical methods. Although all three studies expressed their effect sizes using odds ratio values, one of the studies also conducted a mediation analysis.

**Green space and nervous system diseases**

Of the eight studies included in this systematic review of nervous system diseases, only three had data that could be combined. The remaining studies had fewer than three outcome indicators each, making it impossible to conduct a meta-analysis. The other five studies all focused on the impact of green space exposure on the cognition of the elderly. However, there was significant heterogeneity in the outcome indicators. One study focused on the mortality rate of neurodegenerative diseases^[41]^, two studies focused on the incidence of dementia^[37,49]^, one study focused on brain MRI indicators^[32]^, and one study focused on the decline in cognitive function as measured by the Mini-Mental State Examination score^[31]^. At the same time, the five studies also differed significantly in the following aspects:

Population characteristics: Three studies were from the United States and had a predominantly Caucasian population, one study was from China, and one study was from Belgium. There were also differences in age structures: Three studies had a population range of ≥65 years old, one study was ≥60 years old, and one study was ≥75 years old.

Measurement of green space exposure: All five studies used NDVI to assess exposure levels, but the set buffer radius ranged from 300 to 5,000 meters.

Statistical methods: The effect sizes were not uniform. Two studies used hazard ratio (HR), two studies used odds ratio (OR), and one study used β coefficient.

**Green space and mental illness**

Of the seven studies included on the impact of green space exposure on the mental health of older adults. We combined the data from three of the seven studies on the impact of green space exposure on the mental health of older adults. The remaining four studies had different outcomes (three measured depression ^[39, 42, 43]^), study designs (two were cross-sectional study and one was a cohort study), and populations (Two studies were conducted targeting people aged 60 and older in China. One study recruited community-dwelling older adults from the general population, and the other focused on older adults covered by Shanghai's long-term care insurance program. A third study recruited retired individuals aged 65 and older living in urban or suburban areas in the United States.). The dimensions of exposure measurement differed as well: all three studies used the NDVI to quantify green space exposure. However, they employed different buffer radii. One study used 250m and 1,000m buffers. Another study used a fixed 1,000-meter buffer. The third study used concentric 100m, 300m, and 500m buffers. There was heterogeneity in these aspects. We were unable to perform a meta-analysis on another mental health study because it did not meet the requirement of having more than three outcome measures.

**Green space and life satisfaction**

The two studies^[46,53]^ we included on the impact of green space exposure on life satisfaction among older adults did not meet the requirement of having more than three identical outcome indicators. Therefore, we were unable to conduct a meta-analysis. The two studies have significant differences in their research populations (one is from Malaysia and the other is from China) and in their measurement of outcome indicators (one uses the Well-Being of Older People Scale and the other uses the Satisfaction with Life Scale). These two scales have different measurement dimensions for outcome indicators, so they are not comparable. Additionally, the two studies have different measurement dimensions for exposure indicators. One study focuses on the spatial scale of green space exposure, such as area, vegetation richness, and facilities. The other emphasizes accessibility indicators of green space, such as green space rate and distance to the nearest park.
